# Machine Learning Consensus Clustering Approach for Hospitalized Patients with Dysmagnesemia

**DOI:** 10.3390/diagnostics11112119

**Published:** 2021-11-15

**Authors:** Charat Thongprayoon, Janina Paula T. Sy-Go, Voravech Nissaisorakarn, Carissa Y. Dumancas, Mira T. Keddis, Andrea G. Kattah, Pattharawin Pattharanitima, Saraschandra Vallabhajosyula, Michael A. Mao, Fawad Qureshi, Vesna D. Garovic, John J. Dillon, Stephen B. Erickson, Wisit Cheungpasitporn

**Affiliations:** 1 Division of Nephrology and Hypertension, Department of Medicine, Mayo Clinic, Rochester, MN 55905, USA; charat.thongprayoon@gmail.com (C.T.); sy-go.janina@mayo.edu (J.P.T.S.-G.); dumancas.carissa@mayo.edu (C.Y.D.); kattah.andrea@mayo.edu (A.G.K.); Qureshi.Fawad@mayo.edu (F.Q.); garovic.Vesna@mayo.edu (V.D.G.); dillon.John@mayo.edu (J.J.D.); erickson.stephen@mayo.edu (S.B.E.); 2 Division of Nephrology, Department of Medicine, Beth Israel Deaconess Medical Center, Harvard Medical School, Boston, MA 01702, USA; voravech.niss@gmail.com; 3 Division of Nephrology and Hypertension, Department of Medicine, Mayo Clinic, Phoenix, AZ 85054, USA; keddis.Mira@mayo.edu; 4 Department of Internal Medicine, Faculty of Medicine, Thammasat University, Pathum Thani 12121, Thailand; pattharawin@hotmail.com; 5 Section of Cardiovascular Medicine, Department of Medicine, Wake Forest University School of Medicine, Winston-Salem, NC 27101, USA; svallabh@wakehealth.edu; 6 Division of Nephrology and Hypertension, Department of Medicine, Mayo Clinic, Jacksonville, FL 85054, USA; mao.michael@mayo.edu

**Keywords:** artificial intelligence, clustering, consensus clustering, dysmagnesemia, electrolytes, hypomagnesemia, hypermagnesemia, individualized medicine, machine learning, magnesium, mortality, nephrology, personalized medicine, precision medicine

## Abstract

Background: The objectives of this study were to classify patients with serum magnesium derangement on hospital admission into clusters using unsupervised machine learning approach and to evaluate the mortality risks among these distinct clusters. Methods: Consensus cluster analysis was performed based on demographic information, principal diagnoses, comorbidities, and laboratory data in hypomagnesemia (serum magnesium ≤ 1.6 mg/dL) and hypermagnesemia cohorts (serum magnesium ≥ 2.4 mg/dL). Each cluster’s key features were determined using the standardized mean difference. The associations of the clusters with hospital mortality and one-year mortality were assessed. Results: In hypomagnesemia cohort (*n* = 13,320), consensus cluster analysis identified three clusters. Cluster 1 patients had the highest comorbidity burden and lowest serum magnesium. Cluster 2 patients had the youngest age, lowest comorbidity burden, and highest kidney function. Cluster 3 patients had the oldest age and lowest kidney function. Cluster 1 and cluster 3 were associated with higher hospital and one-year mortality compared to cluster 2. In hypermagnesemia cohort (*n* = 4671), the analysis identified two clusters. Compared to cluster 1, the key features of cluster 2 included older age, higher comorbidity burden, more hospital admissions primarily due to kidney disease, more acute kidney injury, and lower kidney function. Compared to cluster 1, cluster 2 was associated with higher hospital mortality and one-year mortality. Conclusion: Our cluster analysis identified clinically distinct phenotypes with differing mortality risks in hospitalized patients with dysmagnesemia. Future studies are required to assess the application of this ML consensus clustering approach to care for hospitalized patients with dysmagnesemia.

## 1. Introduction

Magnesium is one of the most common cations in the body with great physiologic importance, including signal transduction, glycolysis, oxidative phosphorylation, protein synthesis and degradation, and more than 300 intracellular reactions [1,2,3,4,5,6,7]. Previous studies have reported hypomagnesemia and hypermagnesemia as common as 11–20% and 9–12%, respectively [8,9]. Dysregulated magnesium level or dysmagnesemia, whether hypomagnesemia [8,10,11,12] or hypermagnesemia [8,11,12,13,14], has been associated with adverse outcomes, including cardiac arrhythmias, respiratory failure, and increased mortality in various patient populations [8,11,13,15,16,17,18,19]. Magnesium homeostasis is regulated by intestinal absorption and renal excretion of magnesium [20], and identifying the phenotype of patients with dysmagnesemia on hospital admission can often be challenging because of lack of detailed clinical information prior to admission and limited data on urinary magnesium.

With the advancement of electronic medical record (EMR) and artificial intelligence, machine learning (ML) approaches have been developed as part of precision medicine to assist in clinical decision-making, including disease detection, medical imaging, and explainable risk prediction [21,22,23,24,25,26,27,28,29]. In recent years, unsupervised ML algorithms have been utilized to reveal the patterns of diseases such as diabetes and cardiovascular diseases [30,31,32,33]. Consensus clustering is an unsupervised ML technique used to identify patterns of data, and provides a visualization tool to inspect cluster numbers, membership, and boundaries [34]. It can be utilized to search for similarities and heterogeneities among data and isolate them into clinically meaningful clusters [22,35]. Recent investigations have demonstrated that ML clustering methods can distinguish meaningful disease subtypes associated with different clinical outcomes [36,37]. Given the heterogeneity of patients with dysmagnesemia on hospital admission [8], the application of ML consensus clustering may help identify distinct phenotypic and clinicopathological clusters of dysmagnesemia that are associated with different clinical outcomes.

In this study, we aimed to identify clinically meaningful clusters of hospitalized patients with dysmagnesemia on hospital admission using an unsupervised ML approach and to assess mortality risks among these distinct clusters. 

## 2. Methods

### 2.1. Patient Population

Adult patients (age ≥ 18 years) admitted to Mayo Clinic in Rochester, Minnesota, USA from January 2009 to 31 December 2013 were screened. Patients with serum magnesium outside the normal reference range (1.7–2.3 mg/dL) on hospital admission were included. Patients who did not have serum magnesium measurement within 24 h of hospital admission or had normal serum magnesium on hospital admission were excluded. Patients were divided into 2 cohorts: (1) hypomagnesemia cohort (serum magnesium ≤ 1.6 mg/dL) and (2) hypermagnesemia cohort (serum magnesium ≥ 2.4 mg/dL). The Mayo Clinic Institutional Review Board approved this study (IRB number 21-003088 and date of approval; 30 March 2021). All included patients provided research authorization. 

### 2.2. Data Collection

As previously described, we collected pertinent demographic information, principal diagnoses, comorbidities, and laboratory data from our hospital’s EMR [8,14]. Only available data within 24 h of hospital admission were incorporated into cluster analysis. If there were multiple laboratory values, the first one within the 24-h time frame was used. We excluded variables with more than 10% missing data. If the variable had missing data less than 10%, the missing data were imputed using Random Forest multiple imputation technique before inputting the data into cluster analysis [38]. 

### 2.3. Cluster Analysis

Unsupervised ML consensus clustering analysis was applied to identify clinical clusters of hypomagnesemia and hypermagnesemia cohorts [39]. We utilized a pre-specified subsampling parameter of 80% with 100 iterations. The number of possible clusters (k) was selected to be between 2 to 10 in order to avoid excessive numbers of clusters that would not be clinically useful. The ideal number of clusters was ascertained by evaluating the cumulative distribution function (CDF), consensus matrix (CM) heat map, cluster-consensus plots in the within-cluster consensus scores, and the proportion of ambiguously clustered pairs (PAC) [34,40]. The within-cluster consensus score (range 0–1) is defined as the average consensus value for all pairs of individuals belonging to the same cluster [34]. A value closer to one indicates better cluster stability [34]. PAC (range 0–1) is calculated as the proportion of all sample pairs with consensus values falling within the predetermined boundaries [40]. A value closer to 0 signifies higher cluster stability [40]. The details regarding the consensus cluster algorithms can be found in the online supplementary.

### 2.4. Statistical Analysis

After cluster identification, subsequent analyses were performed to characterize differences among the clusters. Clinical characteristics between the clusters were compared using Student’s *t*-test for continuous variables and Chi-squared test for categorical variables. The key features of each cluster were determined using the standardized mean difference in clinical characteristics between each cluster and the overall cohort, and clinical characteristics with absolute standardized mean difference of >0.3 were included. Hospital mortality and one-year mortality were compared among the clusters. Logistic regression was used to assess the association of the cluster with hospital mortality, and odds ratio (OR) with 95% confidence interval (95% CI) was reported. In contrast, Cox proportional hazard regression was used to assess the association of the cluster with one-year mortality, and hazard ratio (HR) with 95% CI was reported. We did not adjust for differences in clinical variables between groups because these variables were utilized through unsupervised machine learning to identify the clusters. We used the ConsensusClusterPlus package (version 1.46.0) for consensus clustering analysis, and the “missForest” package for missing data imputation [41]. We used R, version 4.0.3 (RStudio, Inc., Boston, MA, USA) for all analyses.

## 3. Results

### 3.1. Hypomagnesemia Cohort

There were 65,974 hospitalized patients with available admission serum magnesium measurement. A total of 13,320 (20%) patients presented with hypomagnesemia on hospital admission. The mean age was 61 ± 17 years. 47% were male. The mean estimated glomerular filtration rate (eGFR) was 76 ± 31. The mean admission serum magnesium was 1.5 ± 0.2 mg/dL.

**Figure 1:**
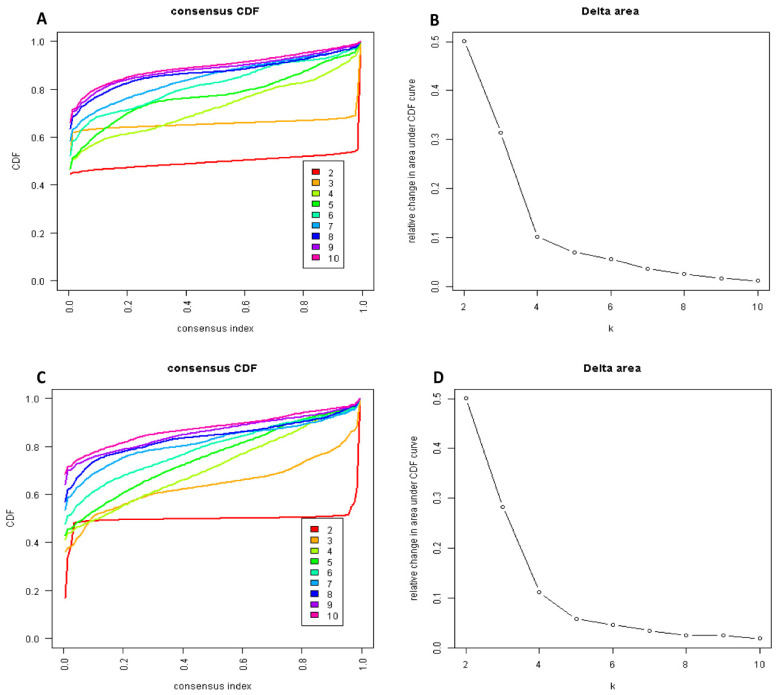
(**A**) Cumulative distribution function (CDF) plot displaying consensus distributions for each cluster (k) for patients with hypomagnesemia; (**B**) Delta area plot reflecting the relative changes in the area under the CDF curve for hypomagnesemia. (**C**) CDF plot displaying consensus distributions for each cluster (k) for patients with hypermagnesemia; (**D**) Delta area plot reflecting the relative changes in the area under the CDF curve for hypermagnesemia.

**Figure 2:**
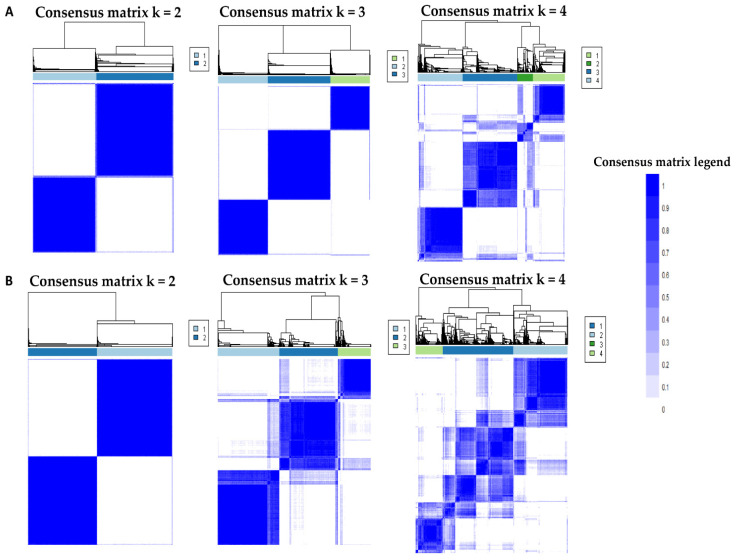
(**A**) Consensus matrix heat map depicting consensus values on a white to blue color scale of each cluster (k) for patients with hypomagnesemia; (**B**) Consensus matrix heat map depicting consensus values on a white to blue color scale of each cluster (k) for patients with hypermagnesemia.

**Figure 3:**
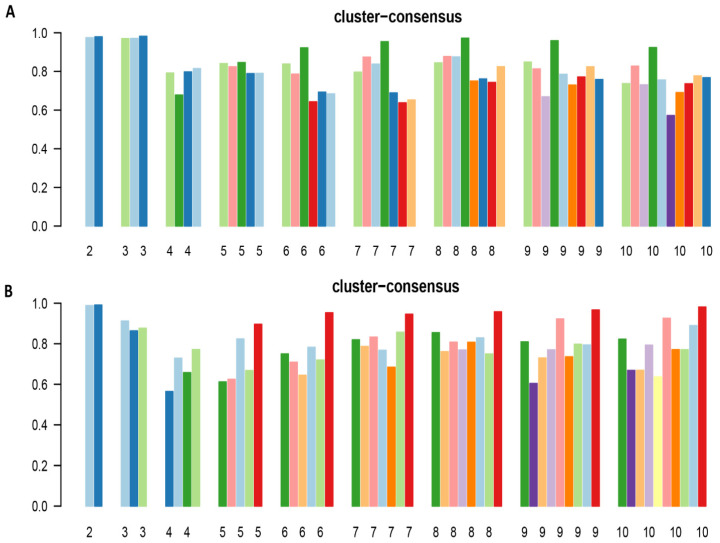
(**A**) The bar plot represents the mean consensus score for different numbers of clusters (K ranges from two to ten) for patients with hypomagnesemia; (**B**) The bar plot represents the mean consensus score for different numbers of clusters (K ranges from two to ten) for patients with hypermagnesemia.

The CDF plot displays the consensus distributions for each hypomagnesemia cluster (Figure 1A, Supplementary Figure S1). The delta area plot, in turn demonstrates the relative change in the area under the CDF curve (Figure 1B, Supplementary Figure S2). The largest changes in area occurred between k = 2 and k = 4. Beyond this range, the relative increase in area became significantly smaller. The CM heatmap (Figure 2A, Supplementary Figures S3–S11) reveals that the ML algorithm identified k = 2 and 3 with clear boundaries (Figure 2A), indicating good cluster stability over repeated iterations. K = 2 and 3 also had high stability given their high mean cluster consensus score (Figure 3A). K = 3 exhibited favorably low PACs (Supplementary Figure S12); Thus, the consensus clustering analysis from available hospital admission baseline characteristics identified three clusters that best represented the data pattern of our patients admitted with hypomagnesemia.

Cluster 1 had 3446 (26%) patients. Cluster 2 had 4351 (33%) patients. Cluster 3 had 5523 (41%) patients. As shown in Table 1, baseline characteristics significantly differed among the three clusters in the hypomagnesemia cohort. 

**Figure 4:**
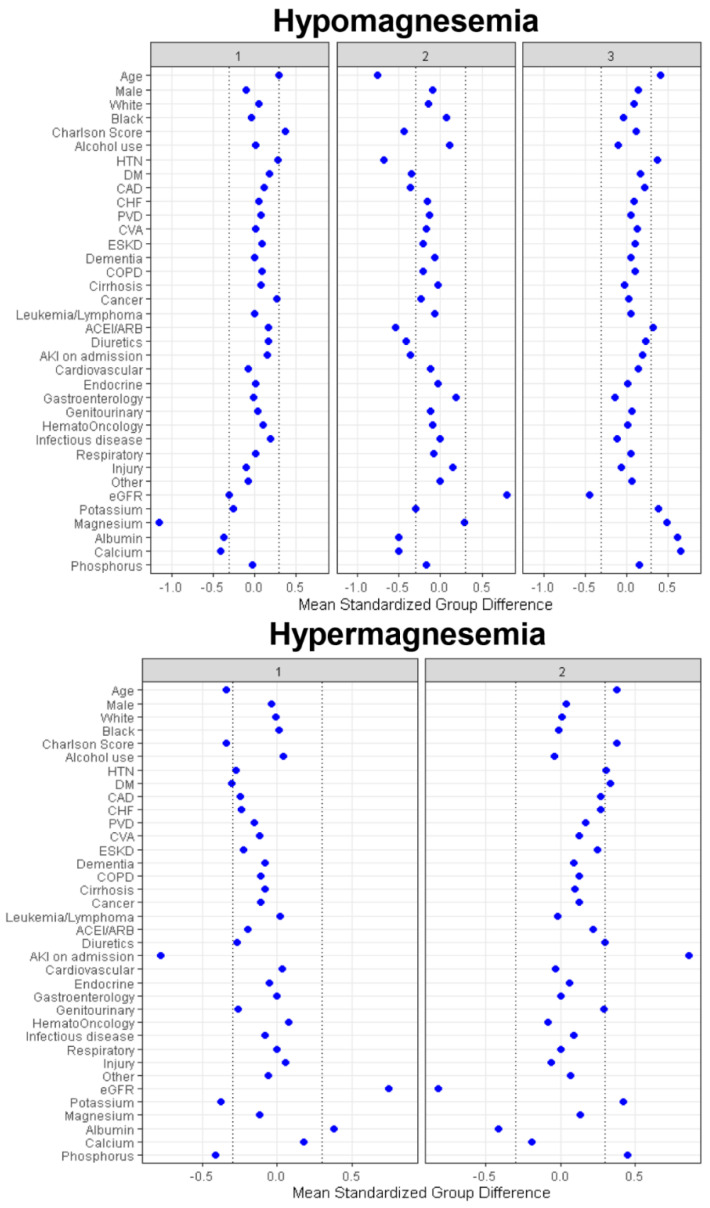
The standardized differences across three clusters for each of baseline parameters for patients with hypomagnesemia and hypermagnesemia. The *x*-axis represents the standardized differences value, and the y axis represents baseline variables. The dashed vertical lines signify the standardized differences cutoffs of <−0.3 or >0.3. Abbreviations: AG, anion gap; AKI, acute kidney injury; BMI, body mass index; CHF, congestive heart failure; Cl, chloride; COPD, chronic obstructive pulmonary disease; CVA, cerebrovascular accident; DM, diabetes mellitus; ESKD, end stage kidney disease; GFR, glomerular filtration rate; GI, gastrointestinal; Hb, hemoglobin; HCO3, bicarbonate; K, potassium; ID, infectious disease; MI, myocardial infarction; Na, sodium; PVD, peripheral vascular disease; RS, respiratory system; SID, strong ion difference.

Based on standardized mean difference shown in Figure 4, cluster 1 was mainly characterized by higher comorbidity burden and lower serum magnesium, albumin, and calcium. On the other hand, cluster 2 was mainly characterized by younger age, lower comorbidity burden, especially less history of hypertension, diabetes mellitus, coronary artery disease, and leukemia/lymphoma, less use of angiotensin converting enzyme inhibitors (ACEI)/angiotensin receptor blockers (ARB), and diuretics, less acute kidney injury (AKI), higher eGFR, and lower serum albumin and calcium. Lastly, cluster 3 was mainly characterized by older age, more history of hypertension, more use of ACEI/ARB, lower eGFR, higher serum potassium, magnesium, albumin, and calcium. 

**Figure 5:**
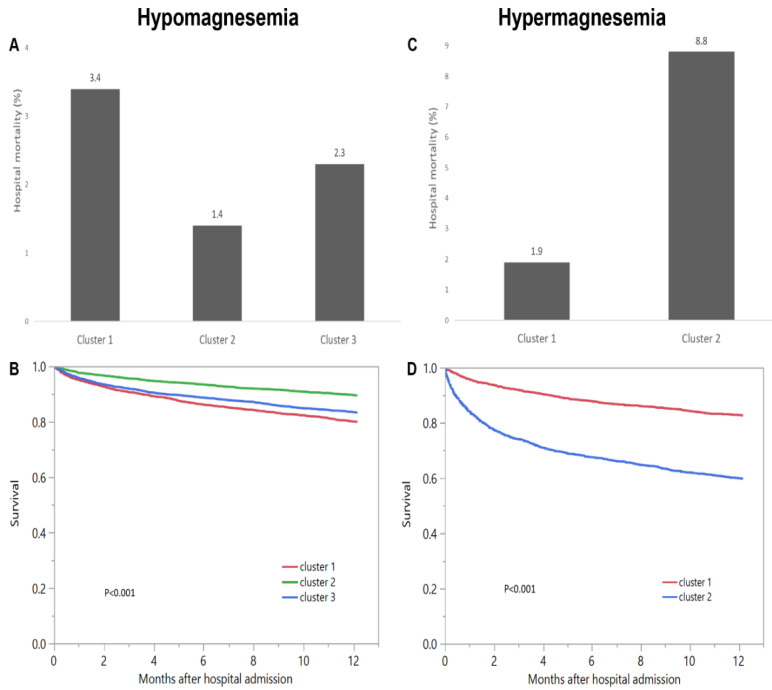
(**A**) Hospital mortality and (**B**) One-year mortality among different clusters of admission hypomagnesemia; (**C**) Hospital mortality and (**D**) One-year mortality among different clusters of admission hypermagnesemia.

The hospital-mortality was 3.4% in cluster 1, 1.4% in cluster 2, and 2.3% in cluster 3 (<0.001) (Figure 5A). Compared to cluster 2, cluster 1 and cluster 3 had higher odds of hospital mortality with OR of 2.45 (95% CI 1.79–3.35), and 1.63 (95% CI 1.20–2.22) respectively. The one-year mortality was 20.1% in cluster 1, 10.5% in cluster 2, and 16.8% in cluster 3 (Figure 5B). 

Compared to cluster 2, cluster 1 and cluster 3 also had higher risk of one-year mortality with HR of 2.04 (95% CI 1.79–2.32) and 1.68 (95% CI 1.49–1.90) respectively (Table 2).

### 3.2. Hypermagnesemia Cohort

A total of 4671 (7%) patients presented with hypermagnesemia on hospital admission. The mean age was 65 ± 17 years. 61% were male. The mean eGFR was 56 ± 34. The mean admission serum magnesium was 2.6 ± 0.4 mg/dL.

The CDF plot displays the consensus distributions for each hypermagnesemia cluster (Figure 1C, Supplementary Figure S13). The delta area plot, in turn demonstrates the relative change in the area under the CDF curve (Figure 1D, Supplementary Figure S14). The largest changes in area occurred between k = 2 and k = 4. Beyond this range, the relative increase in area became significantly smaller. The CM heatmap (Figure 2B, Supplementary Figures S15–S23) reveals that the ML algorithm identified k = 2 with clear boundaries (Figure 2B), indicating good cluster stability over repeated iterations. K = 2 also had high stability given its high mean cluster consensus score (Figure 3B). Favorably low PACs were demonstrated for k = 2 (Supplementary Figure S24). Thus, the consensus clustering analysis from available hospital admission baseline characteristics identified two clusters that optimally represented the data pattern of our patients admitted with hypermagnesemia. 

Cluster 1 had 2445 (52%) patients while cluster 2 had 2226 (48%) patients. As shown in Table 1, clinical characteristics were significantly different between the two identified clusters in the hypermagnesemia cohort. Based on standardized mean difference shown in Figure 4, the key features of patients in cluster 2, compared to those of patients in cluster 1, included older age, higher comorbidity burden, especially more history of hypertension and diabetes mellitus, more AKI, more hospital admissions primarily due to kidney disease, and lower eGFR and serum albumin but higher potassium and phosphorus. 

Cluster 1 had hospital mortality of 1.9%, while cluster 2 had hospital mortality of 8.8% (*p* < 0.001) (Figure 5C). Compared to cluster 1, cluster 2 had higher odds of hospital mortality with OR of 5.04 (95% CI 3.63–6.98). Cluster 1 had one-year mortality of 17.3%, whereas cluster 2 had one-year mortality of 40.3% (*p* < 0.001) (Figure 5D). Compared to cluster 1, cluster 2 also had higher risk of one-year mortality with HR of 2.85 (95% CI 2.51–3.24) (Table 2).

## 4. Discussion

ML consensus clustering algorithms offer the ability to efficiently analyze and identify clusters of patients with different characteristics in a large amount of data [22,35,42,43]. In this study, the unsupervised ML consensus clustering approach was utilized to distinguish patients with dysmagnesemia into distinct clusters. Among patients with hypomagnesemia on hospital admission, age, comorbidity burden, kidney function (with baseline eGFR and AKI on admission as surrogate markers), and degree of hypomagnesemia were important features to differentiate the phenotypes. Similarly, among patients with hypermagnesemia on hospital admission, age, comorbidity burden, kidney function, and principal genitourinary diagnosis were important features to differentiate the phenotypes.

Applying the unsupervised consensus clustering approach to the patient characteristics at the time of hospital admission, we identified three clinically distinct clusters of patients with concomitant hypomagnesemia. The three clusters demonstrated different characteristics and were associated with different hospital and one-year mortality risks. Even though the majority of the patients across all three clusters presented with similar conditions (mainly hematology/oncology-, cardiovascular-, and gastrointestinal-related conditions), the three clusters demonstrated different clinical outcomes. 

Cluster 2, the reference cluster of hypomagnesemia, consisted of patients with younger age with the lowest comorbidity burden compared to other clusters. They also had higher eGFR, lower use of ACEI/ARB and diuretics, and lower incidence of AKI. Interestingly, they had the highest prevalence of alcohol use. They also had the lowest serum albumin, calcium, and phosphate compared to other clusters. Calcium and magnesium are electrolytes that normally bind to albumin. Alteration of circulating albumin levels alters the measured levels of these electrolytes. Measured or total calcium and magnesium alike are lower in the setting of concomitant low serum albumin [44,45,46]. In addition, it is possible that alcoholism and malnutrition played important roles in the development of hypomagnesemia in this patient population because of poor oral intake. Furthermore, these patients also had the highest principal diagnosis of gastrointestinal conditions on admission, which could have potentially resulted in gastrointestinal magnesium loss or redistribution of magnesium triggered by acute pancreatitis [47,48]. Given the fact that patients in cluster 2 were younger and had the lowest comorbidity burden, they had the lowest in-hospital and one-year mortality risks among the three clusters.

Compared to the patients in cluster 2 of hypomagnesemia, those in clusters 1 and 3 were older and had higher comorbidity burden, reduced kidney function (lower baseline eGFR and higher incidence of AKI), and higher use of ACEI/ARB and diuretics. Patients in cluster 1 had the highest comorbidity burden and the highest prevalence of cancer among the three clusters. They also presented more frequently with a principal diagnosis of infectious disease on hospital admission. Furthermore, they had the lowest serum magnesium. A number of cancer-specific therapies can cause hypomagnesemia via renal magnesium wasting, including platinum-based chemotherapy, anti-epidermal growth factor receptor (EGFR) monoclonal antibodies, inhibitors of human epidermal growth factor receptor 2 (HER2), and calcineurin inhibitors [49]. In addition, cancer patients frequently use medications that cause or exacerbate hypomagnesemia, such as proton pump inhibitors (PPIs), diuretics, and chemotherapy [49]. These patients in cluster 1 had the highest in-hospital and one-year mortality risks among the three clusters, which could potentially be due to poor outcomes among cancer patients with hypomagnesemia [8,50]. Previous studies also reported that hypomagnesemia is associated with increased mortality risk among patients with infection [10,51,52], which is one of the main characteristics of the patients in this cluster.

Patients in cluster 3 of hypomagnesemia were the oldest among the three clusters. They had the highest prevalence of hypertension, coronary artery disease, congestive heart failure, and principal diagnosis of cardiovascular disease on hospital admission. They also had the highest use of diuretics, which could lead to urinary magnesium wasting. Previous studies have shown that hypomagnesemia in patients with cardiovascular diseases carries a high mortality rate [53,54]. While these patients in cluster 3 had increased in-hospital mortality and one-year mortality compared to those in cluster 2, they had lower mortality when compared to those in cluster 1 (phenotype of cancer patients with hypomagnesemia) despite having the oldest age group among the three clusters. 

Within the cohort of hypermagnesemia on hospital admission, we identified two clinically distinct clusters by ML consensus clustering approach. Compared to cluster 1, the key features of cluster 2 included older age, higher comorbidity burden, more hospital admissions primarily due to kidney disease, more AKI, and lower eGFR. Because of the reduction in kidney function, these patients in cluster 2 likely had reduced ability to renally excrete magnesium, resulting in hypermagnesemia. Reported clinical cases include the administration of antacids or magnesium supplements in older patients or those with reduced kidney function [55]. Conversely, patients in cluster 1 were younger but had higher alcohol use and were more likely to be admitted with principal diagnoses of injury/poisoning and hematology/oncology. As such, it is possible that hypermagnesemia among patients in cluster 1 could have resulted from excessive tissue injury or breakdown (such as tumor lysis syndrome and burn injury) [56,57]. Compared to those in cluster 1, patients in cluster 2 had higher hospital mortality and one-year mortality.

The strengths of this study include a large sample size and unbiased data manipulation, and easy reproducibility by unsupervised ML consensus clustering. There are also, however, limitations that should be noted. First, the data were abstracted from a single-center, and our patient population was predominantly Caucasian, which might limit the extrapolation of our findings to other populations. Second, consensus clustering was performed on hospital admission and did not include data before or during hospitalization, which could affect hospitalization-related outcomes. Third, we did not have data on magnesium supplements or other medications that might have affected serum magnesium, such as antibiotics, proton pump inhibitors, and chemotherapy. Fourth, some relevant laboratory results were not available, including 24-h urinary magnesium, fractional urinary excretion of magnesium, and genetic testing (for conditions that might have caused hypomagnesemia in adults, such as Gitelman syndrome). These investigations are not commonly performed on hospital admission and were thus not included in our ML clustering algorithm. Therefore, future studies are required to assess whether these variables could have improved the discriminatory ability of these clusters we identified. Nevertheless, we included readily available data at the time of hospital admission and successfully identified distinct clusters of dysmagnesemia associated with different clinical outcomes. 

## 5. Conclusions

In summary, we present an unsupervised ML consensus clustering analysis of hospitalized patients with dysmagnesemia. We discovered three distinct phenotypes of admission hypomagnesemia and two distinct phenotypes of admission hypermagnesemia with different hospital and one-year mortality risks. With the advancement of EMR and artificial intelligence, future studies are required to evaluate and validate the application of this ML consensus clustering approach to care for hospitalized patients with dysmagnesemia.

## Figures and Tables

**Table 1:** Clinical characteristics.

Patient Characteristics	Hypomagnesemia Cohort (*n* = 13,320)				Hypermagnesemia Cohort (*n* = 4671)		
	Cluster 1	Cluster 2	Cluster 3	*p*-Value	Cluster 1	Cluster 2	*p*-Value
	(*n* = 3446)	(*n* = 4351)	(*n* = 5523)		(*n* = 2445)	(*n* = 2226)	
Age (years)	66.0 ± 13.5	48.2 ± 15.9	67.9 ± 13.4	<0.001	59.5 ± 17.4	71.9 ± 14.6	<0.001
Male sex	1458 (42)	1848 (42)	2987 (54)	<0.001	1444 (59)	1399 (63)	0.008
Race				<0.001			0.72
-White	3208 (93)	3810 (88)	5196 (94)		2198 (90)	2016 (91)	
-Black	40 (1)	109 (3)	64 (1)		53 (2)	43 (2)	
-Others	198 (6)	432 (10)	263 (5)		194 (8)	167 (8)	
Principal diagnosis				<0.001			<0.001
-Cardiovascular	408 (12)	443 (10)	1084 (20)		863 (35)	718 (32)	
-Endocrine/metabolic	133 (4)	136 (3)	218 (4)		83 (3)	126 (6)	
-Gastrointestinal	433 (13)	829 (19)	466 (8)		247 (10)	231 (10)	
-Genitourinary	184 (5)	94 (2)	332 (6)		16 (0.7)	335 (15)	
-Hematology/oncology	1163 (34)	1082 (25)	1632 (30)		283 (12)	157 (7)	
-Infectious disease	334 (10)	226 (5)	159 (3)		67 (3)	141 (6)	
-Respiratory	131 (4)	85 (2)	254 (5)		152 (6)	139 (6)	
-Injury/poisoning	363 (11)	828 (19)	649 (12)		293 (12)	187 (8)	
-Other	297 (9)	628 (14)	729 (13)		441 (18)	192 (9)	
Charlson Comorbidity Score	3.4 ± 3.0	1.3 ± 1.9	2.7 ± 2.6	<0.001	1.4 ± 1.9	3.2 ± 2.8	<0.001
Comorbidities							
-Hypertension	2400 (70)	944 (22)	4101 (74)	<0.001	1088 (44)	1621 (73)	<0.001
-Diabetes mellitus	1169 (34)	455 (10)	1835 (33)	<0.001	282 (12)	862 (39)	<0.001
-Coronary artery disease	788 (23)	190 (4)	1493 (27)	<0.001	451 (18)	933 (42)	<0.001
-Congestive heart failure	215 (6)	67 (2)	398 (7)	<0.001	203 (8)	614 (28)	<0.001
-Peripheral vascular disease	170 (5)	50 (1)	256 (5)	<0.001	49 (2)	204 (9)	<0.001
-Stroke	240 (7)	96 (2)	547 (10)	<0.001	164 (7)	312 (14)	<0.001
-End-stage kidney disease	278 (8)	38 (1)	458 (8)	<0.001	19 (0.8)	270 (12)	<0.001
-Dementia	36 (1)	14 (0.3)	80 (1)	<0.001	26 (1)	79 (4)	<0.001
-COPD	410 (12)	136 (3)	676 (12)	<0.001	232 (9)	393 (18)	<0.001
-Cirrhosis	178 (5)	132 (3)	187 (3)	<0.001	58 (2)	132 (6)	<0.001
-Cancer	1699 (49)	1087 (25)	2071 (37)	<0.001	414 (17)	597 (27)	<0.001
-Leukemia/lymphoma	142 (4)	123 (3)	293 (5)	<0.001	117 (5)	90 (4)	0.22
Alcohol use	234 (7)	393 (9)	213 (4)	<0.001	162 (7)	107 (5)	0.008
Laboratory test							
-eGFR (mL/min/1.73 m^2^)	67 ± 29	101 ± 23	63 ± 26	<0.001	81 ± 25	28 ± 16	<0.001
-Potassium (mEq/L)	3.9 ± 0.6	3.9 ± 0.5	4.3 ± 0.6	<0.001	4.2 ± 0.6	4.8 ± 0.9	<0.001
-Magnesium (mg/dL)	1.3 ± 0.2	1.4 ± 0.1	1.5 ± 0.1	<0.001	2.6 ± 0.4	2.7 ± 0.4	<0.001
-Albumin (g/dL)	3.2 ± 0.5	3.1 ± 0.5	3.7 ± 0.4	<0.001	3.8 ± 0.4	3.4 ± 0.5	<0.001
-Total calcium (mg/dL)	8.3 ± 0.8	8.2 ± 0.6	9.2 ± 0.7	<0.001	9.3 ± 0.6	9.0 ± 0.8	<0.001
-Phosphorus (mg/dL)	3.7 ± 1.0	3.5 ± 0.8	3.8 ± 0.9	<0.001	3.7 ± 0.7	5.0 ± 1.8	<0.001
Medication							
-ACEI/ARB	1629 (47)	557 (13)	3047 (55)	<0.001	789 (32)	1172 (53)	<0.001
-Diuretics	1524 (44)	699 (16)	2602 (47)	<0.001	1007 (41)	1150 (70)	<0.001
Acute kidney injury	830 (24)	179 (4)	1431 (26)	<0.001	156 (6)	1947 (87)	<0.001

**Table 2:** Mortality outcomes according to clusters.

	Hospital Mortality	OR (95% CI)	1-Year Mortality	HR (95% CI)
(a) Hypomagnesemia cohort				
Cluster 1	3.4%	2.45 (1.79–3.35)	20.1%	2.04 (1.79–2.32)
Cluster 2	1.4%	1 (ref)	10.5%	1 (ref)
Cluster 3	2.3%	1.63 (1.20–2.22)	16.8%	1.68 (1.49–1.90)
(b) Hypermagnesemia cohort				
Cluster 1	1.9%	1 (ref)	17.3%	1 (ref)
Cluster 2	8.8%	5.04 (3.63–6.98)	40.3%	2.85 (2.51–3.24)

## Data Availability

Data is available upon reasonable request to the corresponding author.

## Supplementary Materials

The following are available online at https://www.mdpi.com/article/10.3390/diagnostics11112119/s1, Figures S1–S23: Figure S1 and S13, consensus CDF; Figure S2 and S14, relative change in area under CDF curve; Figures S3–S11 and S15–S23, consensus matrix heat map depicting consensus values on a white to blue color scale of each cluster; Figures S12 and S24 PAC.

## Abbreviations

ACEI	angiotensin converting enzyme inhibitors
AG	anion gap
AKI	acute kidney injury
ARB	angiotensin receptor blockers
BMI	body mass index
CDF	cumulative distribution function
CHF	congestive heart failure
CI	confidence interval
Cl	chloride
CM	consensus matrix;
COPD	chronic obstructive pulmonary disease
CVA	cerebrovascular accident
DM	diabetes mellitus
eGFR	estimated glomerular filtration rate
EMR	electronic medical record
ESKD	end stage kidney disease
GI	gastrointestinal
Hb	hemoglobin
HCO3	bicarbonate
HR	hazard ratio
ID	infectious disease
K	potassium
MI	myocardial infarction
ML	machine learning
Na	sodium
OR	odds ratio
PAC	proportion of ambiguously clustered pairs
PVD	peripheral vascular disease
RS	respiratory system
SID	strong ion difference
