# Supplementary material for: Machine Learning Consensus Clustering Approach for Hospitalized Patients with Dysmagnesemia
Source: Diagnostics (Basel). 2021 Nov 15;11(11):2119. doi: 10.3390/diagnostics11112119 (PMC8619519; doi:10.3390/diagnostics11112119)
Supplement: Supplementary file 1 [file diagnostics-11-02119-s001.zip › agronomy-1413519-supplementary.pdf]

Supplemental Information

Supplemental Table S1. Model selection and statistical inferences for each scenario. Model selections were made based on Akaike Information Criterion (AICc). Inferences from the models are based on likelihood ratio tests comparing models with and without the specific predictor variables.

Location	Treatment	Model	Predictor Variable	Df	F	Pr(F)	
			time	1	5.84	0.01	*
	UTC	polynomial	time^2	1	6.13	0.01	*
			time^3	1	4.51	0.03	*
			time	1	9.97	0.002	**
Shuttle	1,3-dichloropropene	quadratic	time^2	1	17.46	<0.001	***
			time^3	1	---	---	---
			time	1	1.59	0.20	
	Aldicarb	quadratic	time^2	1	5.29	0.02	*
			time^3	1	---	---	---
			time	1	16.56	<0.001	***
	UTC	polynomial	time^2	1	19.61	<0.001	***
			time^3	1	18.42	<0.001	***
			time	1	0.54	0.46	
Hamilton	1,3-dicloropropene	linear	time^2	1	---	---	---
			time^3	1	---	---	---
			time	1	6.74	0.01	*
	Aldicarb	polynomial	time^2	1	6.89	0.01	*
			time^3	1	5.96	0.01	*
			time	1	11.14	0.001	**
	UTC	polynomial	time^2	1	10.13	0.001	**
			time^3	1	7.94	0.005	**
			time	1	16.43	<0.001	***
Oswego	1,3-dicloropropene	polynomial	time^2	1	10.76	0.001	**
			time^3	1	5.92	0.01	*
			time	1	2.67	0.10	
	Aldicarb	quadratic	time^2	1	7.34	0.008	**
			time^3	1	---	---	---
